# Profile of para athletes and characterization of sports injuries during the 2023 Paralympic School Games

**DOI:** 10.1080/23335432.2025.2531944

**Published:** 2025-07-16

**Authors:** Ana Flávia Medeiros Ribeiro, Renato de Souza Melo, Daniela Cristina Rodrigues Carvalho, Juliana Fernandes, Saulo Fernandes Melo de Oliveira, Rafaella de Andrade Monteiro, Andressa da Silva de Mello, Caroline de Cássia Batista de Souza, Maria Julia de Lyra Cardoso, Maria Das Graças Rodrigues de Araújo, Ana Paula de Lima Ferreira

**Affiliations:** a Department of Physical Therapy, Federal University of Pernambuco, Recife, Brazil; b Department of Sports in the School of Physical Education, Physiotherapy and Occupational Therapy, Minas Gerais Federal University, Belo Horizonte, Brazil

**Keywords:** Sports injuries, musculoskeletal injuries, para-athletes, para-sports

## Abstract

This article explores the characterization of sports injuries among young athletes participating in the 2023 Paralympic School Games. Injuries during the physiological growth phase can not only compromise the development of sports skills but also harm the physical development expected for their age group. Adaptive mechanisms to training are the main determinants of the location of injuries, traumas and musculoskeletal complaints. This paper aimed to identify occurrence of sports injuries in para athletes during the 2023 School Paralympics. The study population consisted of students who participated in the national stage of the 2023 School Paralympics, held at the Paralympic Training Center in the state of São Paulo. The sample consisted of 640 para-athletes, 253 (60.5%) females and 387 (60.5%) males, with an average age of 14.06 years. Overall, 14.9% of the participants reported injuries/illness. The team with the highest percentage of injured members was from the state of Tocantins (14.8%). Most of the injured athletes had intellectual disabilities (41.46%), practiced athletics (56.9%) and were in the final years of elementary school (21.95%), with an average age of 14.56 years, and the majority were male (56.86%).

## Introduction

Physical activity is vital for children with disabilities, promoting inclusion, social participation and well-being (Reedman et al. 2017). Sports development enhances motor and social skills, supporting rehabilitation (Bratteby Tollerz et al. 2015; Wright et al. 2018; Vancampfort et al. 2021). Parathletics includes adapted sports, while Paralympic sports are high-performance events. The growth of para sports and Paralympic participation reflects increasing popularity (Webborn and Van de Vliet 2012; Fagher et al. 2016, 2019). Paralympic eligibility is based on functional classification, with performance as the key success factor (Keogh 2011; Mann et al. 2021).

Paralympic sports for young athletes are practiced globally, with Brazil playing a key role through the Paralympic School Games organized by the Brazilian Paralympic Committee (CPB) since 2009. The event promotes inclusivity, advancing athletes to national competitions to identify talent for international events (Bataglion and Mazo 2019). Participants develop values like solidarity and fair play, and the Games inspire new para-athletes as they grow in popularity (Corrêa de Resende et al. 2019).

Youth experience higher musculoskeletal stress than adults, increasing the risk of injuries and long-term issues like osteoarthritis and sports abandonment (Myer et al. 2015; Fagher et al. 2019; Monasterio et al. 2024). Children with disabilities face additional risks from muscle weakness, atypical activity patterns, stiffness, poor coordination and compensatory mechanisms (Swain et al. 2018; Fiorese et al. 2020; Rodríguez Macías et al. 2022; Zwierzchowska et al. 2022).

Children with neurological conditions often have low bone density, increasing the risk of stress fractures (Mergler et al. 2016; Lin et al. 2018). Young para-athletes face higher injury risks due to intense training, affecting both performance and daily activities (Fagher et al. 2016, 2022). While research on para-athletes is limited, studies on non-disabled athletes show high injury rates, especially in lower extremities and concussions, with potential growth effects (Reid et al. 2007; Bergeron et al. 2015). The juvenile musculoskeletal system is more prone to trauma, increasing fracture and microtrauma risks (Costa et al. 2022). Injury rates are influenced by training, age and load, with poor training causing strain and inadequate recovery (Jayanthi et al. 2019).

Despite progress in para-athlete research, there is a gap in understanding sports injury epidemiology among young para-athletes, particularly during the Paralympic School Games. Most studies focus on adult or Olympic athletes, overlooking the unique traits of younger athletes, which hinders preventive and treatment strategies. This study aimed to analyze the profile of para-athletes and sports injuries during the 2023 Paralympic School Games.

## Materials and methods

This observational, cross-sectional, descriptive and analytical study followed the STROBE protocol and was conducted during the 2023 Brazilian School Paralympics from November 27 to December 2 at the Paralympic Training Center in São Paulo. It adhered to National Health Council Resolution 466/2012 and the Declaration of Helsinki. All athletes and guardians signed the Free and Informed Consent Form, and the study was approved by the PTC and the Research Ethics Committee of the Federal University of Pernambuco (CAEE: 69652123.8.0000.5208; Report No. 6,273,049).

The sample size was calculated using Derman et al. (2020), which reported a 13.94% sports injury incidence among para-athletes aged 12–25. A 95% confidence interval and 5% margin of error led to a minimum sample of 184. Inclusion followed Brazilian Paralympic Committee guidelines, with exclusion for refusal to sign consent forms. Volunteers were recruited before and after events to avoid disrupting performance.

Two Google Forms questionnaires were created for in-person interviews with para-athletes: one for sociodemographic data and the other based on the Brazilian adaptation of the ‘The Oslo Sports Trauma Research Center – Questionnaire on Health Problems – OSTRC-BR’ (Pimenta et al. 2021). Competitors completed the forms independently or with assistance from two trained researchers. For athletes with visual or intellectual disabilities, questions were verbalized, and responses recorded. After each questionnaire, athletes rated the difficulty as (i) Easy, (ii) Mild or (iii) Hard.

## Results

A total of 640 para-athletes completed the sociodemographic questionnaire, with an average age of 14.06 years (ages 10–18). Of the participants, 253 (39.5%) were female and 387 (60.5%) were male. Among the 274 athletes who answered the injury/illness questionnaire, 41 (14.9%) reported experiencing injury or discomfort during the event. The data also covered the type of disability, education level, school type, sport modality and years of participation in sports as presented in Table 1.

**Table 1.** Sociodemographic data.

	Number of Para-athletes	%
**Gender**		
Female	253	39.5
Male	387	60.5
Total	640	100
**Disability**		
Intellectual	131	20.5
Visual	133	20.8
Physical	376	58.8
Total	640	100
**Education**		
Public School	547	85.5
Private School	93	14.5
Total	640	100
**School Year**		
3rd year of elementary school	5	0.8
4th year of elementary school	11	1.7
5th year of elementary school	18	2.8
6th year of elementary school	80	12.5
7th year of elementary school	118	18.4
8th year of elementary school	77	12
9th year of elementary school	114	17.8
1st year of high school	115	18
2st year of high school	62	9.7
3st year of high school	40	6.3
Total	640	100
**Sports Practiced**		
Athletics	198	30.9
Boccia	30	4.7
3x3 Basketball	14	2.2
Blind Football	17	2.7
Football for People with Cerebral Palsy	25	3.9
Goalball	17	2.7
Weightlifting	13	2
Judo	48	7.5
Swimming	154	24.1
Parabadminton	41	6.4
Table Tennis	36	5.6
Wheelchair Tennis	10	1.6
Sitting Volleyball	37	5.8
Total	640	100
**Duration of Practice**		
Less than 1 year	174	27.2
Between 1–2 years	172	26.9
Between 2–3 years	85	13.3
Between 3–4 years	61	9.5
More than 5 years	148	23.1
Total	640	100

(source: author’s own data)

**Figure 1.**
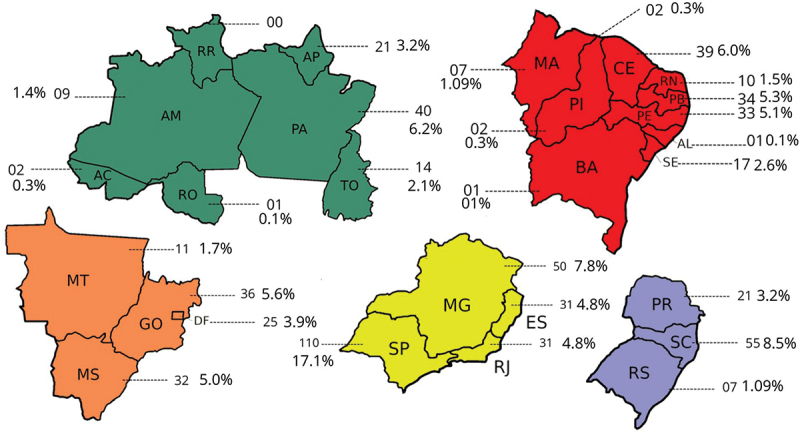
Number of para athletes by state enrolled in the 2023 School Paralympics (*n* = 640). São Paulo/Brazil, 2023 (source: author’s own data).

Notably, out of the 26 states and the Federal District, only the state of Pará was without representation from para-athletes in the competition as shown in Figure 1. The state of São Paulo had the highest participation of athletes *n* = 110 (17.1%), while six states (Acre, Rondônia, Maranhão, Alagoas and Bahia) had the lowest representation with less than 1% of registered athletes each.

**Figure 2.**
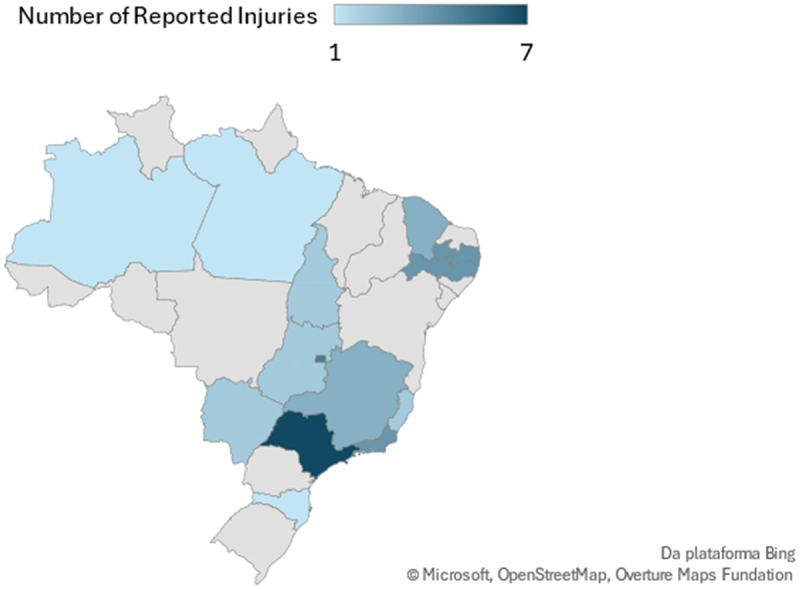
Number of para athletes by state that reported any kind of discomfort, injury or illness at the 2023 School Paralympics (*n* = 41). São Paulo/Brazil, 2023 (source: author’s own data).

**Figure 3.**
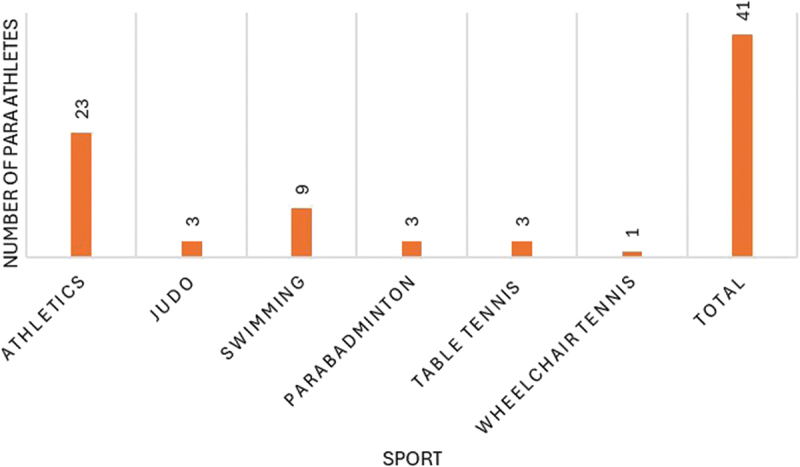
Number of para athletes by sport that reported any kind of discomfort, injury or illness at the 2023 School Paralympics (*n* = 41). São Paulo/Brazil, 2023 (source: author’s own data).

**Figure 4.**
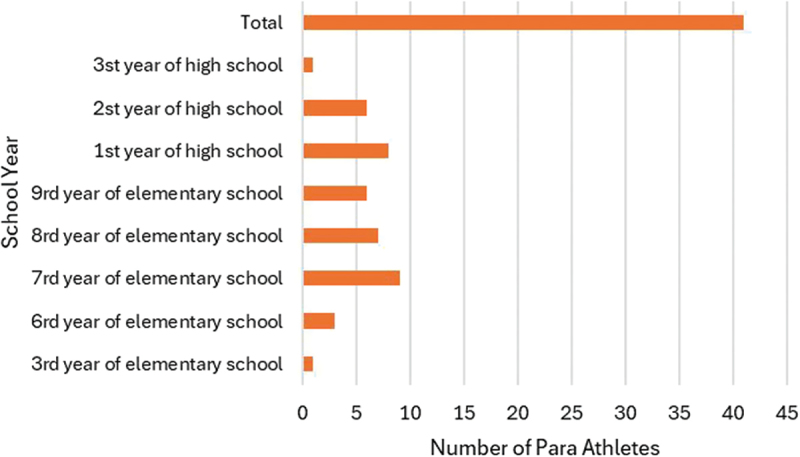
Number of para athletes by school year that reported any kind of discomfort, injury or illness at the 2023 School Paralympics (*n* = 41). São Paulo/Brazil, 2023 (source: author’s own data).

**Figure 5.**
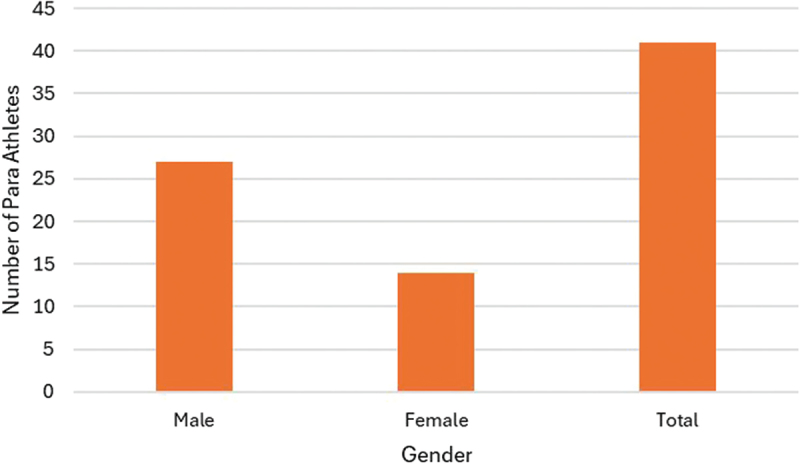
Number of para athletes by gender that reported any kind of discomfort, injury or illness at the 2023 School Paralympics (*n* = 41). São Paulo/Brazil, 2023 (source: author’s own data).

Based on the data collected from the second questionnaire, we are able to profile the participants who reported any form of discomfort, injury or illness, as illustrated by sport, state, gender and school year in the Figures 2, 3, 4 and 5

The number of injuries/illness reported was higher in the State of São Paulo, however when compared by the total number of para athletes in each team, the team of Tocantins had the highest % of the team injured with 14.8%.

Table 2 displays the data gathered from the second questionnaire, such as signs of injuries or illness, specific injury or illnesses, level of participation in competition, reduced training volume, effects on performance, perception of the symptoms/health complaints

**Table 2.** Injury and illness.

	Number of Para-athletes	%
**Signs of injury or illness**		
Yes	41	14.9
No	233	85.1
Total	274	100
**Injury or Illness**		
Injury	38	92.6
Illness	3	7.3
Total	41	100
**Participation in the competition**		
Full participation	9	21.95
Full participation but with difficulty	28	68.29
Reduced participation	3	7.31
Could not participate	1	2.43
Total	41	100
**Reduction in Training**		
No reduction	27	65.85
Small reduction	10	24.39
Moderate reduction	1	2.43
Large reduction	2	4.87
Could not train	1	2.43
Total	41	100
**Reduction in Performance**		
No reduction	12	29.26
Small reduction	15	36.58
Moderate reduction	7	17.07
Large reduction	6	14.63
Could not train	1	2.43
Total	41	100

**Figure 6.**
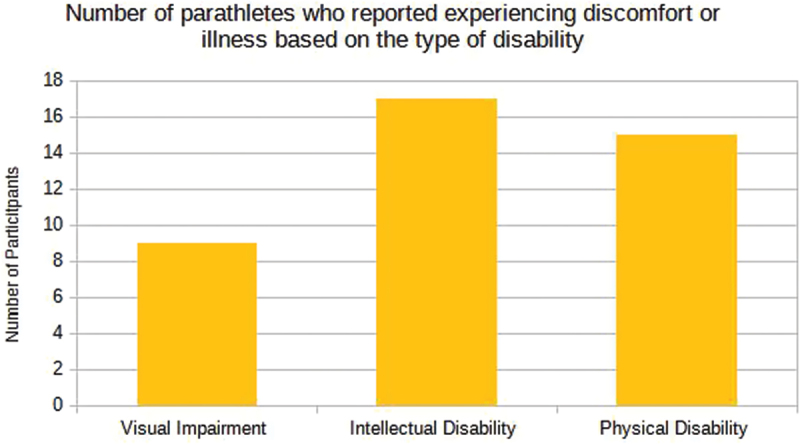
Description of the number of para-athletes who reported experiencing discomfort or illness based on the type of disability presented by the participants in the sample enrolled in the 2023 School Paralympics (*n* = 41), São Paulo/Brazil, 2023. (source: author’s own data).

Of the 41 participants who reported experiencing discomfort, injury or illness during the competition, 9 para athletes have visual impairment (22%), 17 parathletes have intellectual impairment (41.5%), and 15 parathletes have physical impairment (36.6%), as illustrated in Figure 6.

**Figure 7.**
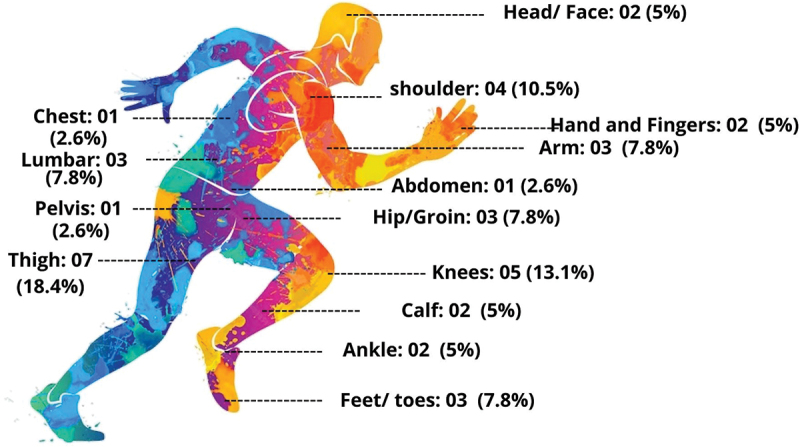
Topography of injuries in the sample of para-athletes enrolled in the 2023 School Paralympics (*n* = 38). São Paulo/Brazil, 2023. (source: author’s own data).

**Figure 8.**
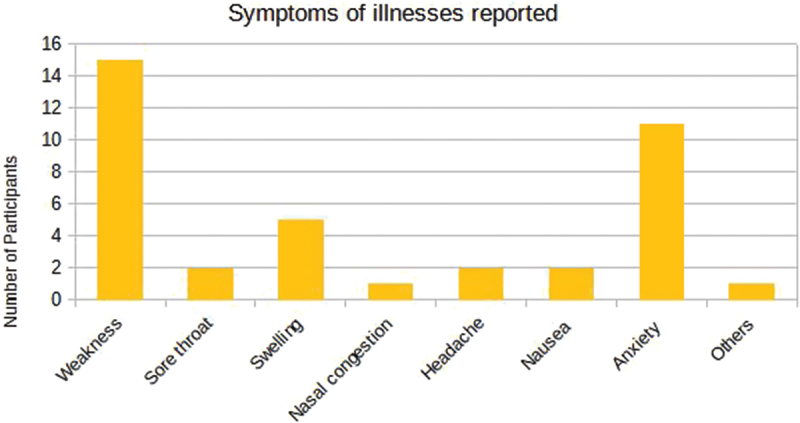
Symptoms of illnesses reported by the sample of para-athletes enrolled in the 2023 School Paralympics (*n* = 39). São Paulo/Brazil, 2023. (source: author’s own data).

Figure 7 shows that some participants reported multiple injury locations along with illness symptoms. The questionnaire data reveals that 39 participants experienced at least one symptom, although only three reported illnesses. Anxiety and weakness were the most common, with 11 (28%) reporting anxiety and 15 (38.4%) reporting weakness, as seen in Figure 8.

**Figure 9.**
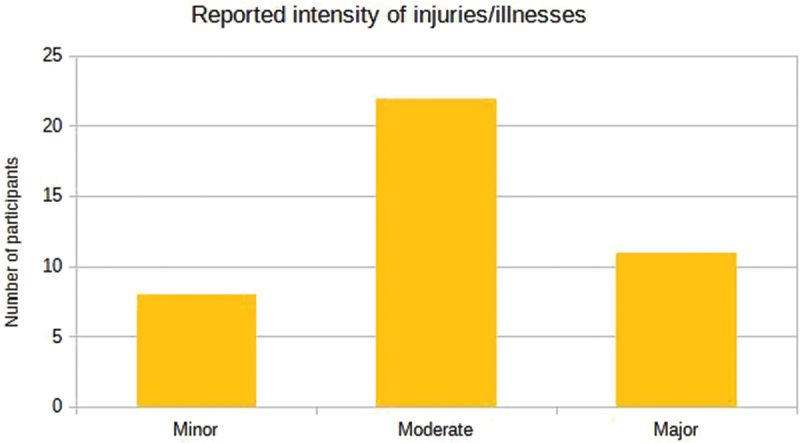
Reported intensity of injuries/illnesses in the sample of para-athletes enrolled in the 2023 School Paralympics (*n* = 41). São Paulo/Brazil, 2023. (source: author’s own data).

**Figure 10.**
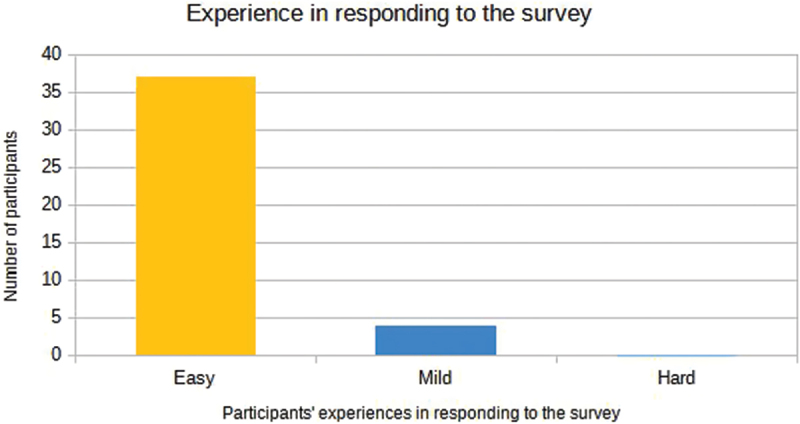
Experience in responding to the survey of participants in the sample enrolled in the 2023 School Paralympics (*n* = 41). São Paulo/Brazil, 2023. (source: author’s own data).

The figures below, Figures 9 and 10, illustrate the results concerning the perceived intensity of the reports, categorized as mild, moderate and severe, as well as the participants’ experiences in responding to the survey, respectively.

Table 3 shows the results of injuries, discomfort or illness reported by participants, considering disability type, education level and sports practice duration. The data reveals a significant relationship between the time spent practicing sports and the occurrence of discomforts (*p* = 0.047).

**Table 3.** Injury and illness by years of practice.

Time in sports	More than 2 years of practice		Less than 2 years of practice		p
	n	%	n	%	
Injuries and Discomforts	28	68.3	13	31.7	0.047*
	Elementary School		High School		0.098
Education level	n	%	n	%	
Injuries and Discomforts	26 63.4		15 36.6		

Test: Pearson Chi-Square.

## Discussion

The aim of this research was to examine the characteristics of para-athletes and the occurrence of sports-related injuries during the 2023 Paralympic School Games. The study found that 85% of participants in the Paralympic School Games were from public schools, reflecting these institutions’ greater ability to create inclusive environments with access to adapted sports. Brazil’s legal frameworks, including the Law for the Inclusion of Persons with Disabilities (Law No. 13146/2015) and National Guidelines for Special Education (CNE/CEB Resolution No. 2/2001), guarantee education rights for all students and require public schools to adapt curricula and train teachers for an inclusive environment.

Pinheiro et al. (2021) conducted a meta-analysis of 30 studies, estimating a 34.7% injury rate among para-athletes. In contrast, our study found that 14.9% of participants (41 individuals) reported injuries during the event. Derman et al. (2018) reported a 13.94% injury rate; the difference may likely be due to the difference in the duration of the studies. The discrepancy in injury rates may also result from the self-reported nature of the questionnaire rather than medical evaluations by health teams or coaches that may underestimate the number of injuries/illness suffered by the participants.

The most reported injuries among para athletes interviewed occurred in the lower limbs (*n* = 23; 59.7%) with the highest concentration of complaints related to the thigh (*n* = 7; 18%) and knee (*n* = 5; 13.1%). These findings align with Hübscher et al. (2010), who identified sprains, dislocations and ligament ruptures, particularly in the knee, as prevalent injuries. Kaeding et al. (2017) also reported that 60% sports-related surgeries in the U.S. are performed on high school athletes with knee issues.

Kaeding et al. (2017) note that sports biomechanics stress static joint structures, increasing knee injury risk. Our study found that para-athletes with intellectual disabilities had the highest injury rates, especially in athletics. Similarly, Tuakli-Wosornu et al. (2018) noted that athletes with intellectual impairments sustain injuries more frequently in track and field than in other sports.

Our study showed a tendency for injuries in para athletes in elementary school, supporting Stracciolini et al. (2017), which found prepubertal children are more prone to repetitive stress injuries due to vulnerable growth cartilage and soft tissues. Wild et al. (2012) noted that the gender gap in injury rates grows around ages 11–12, as puberty increases females’ injury vulnerability.

In our study, anxiety (28%) and weakness (38.4%) were the most common symptoms, with over 50% perceiving their injury/illness as moderate or major. Daley and Reardon (2024) found no clear link between young athletes and mental health disorders but identified issues like depression, burnout and anxiety. Rice et al. (2016) highlighted unique stressors for elite athletes, such as media scrutiny and career-ending injuries, which impact mental health and performance. Many athletes avoid mental health support due to stigma and lack of awareness.

Our study found significant correlations between longer sports practice and higher injury rates, aligning with Warden et al. (2021), Stracciolini et al. (2017) and Myer et al. (2015), who highlight the risks of early sports specialization, including overload injuries, burnout and early dropout, with long-term physical and psychological consequences.

Dahab et al. (2019) report a rise in sports-related injuries among teenage athletes, though the exact causes are unclear. The trend of early sport specialization and intensified training for higher level competition have raised concerns about increased injury risk and negative effects on mental health.

Haraldsdottir and Watson (2021) highlight that injuries can have psychological effects, hindering recovery and increasing reinjury risk. However, research has mostly focused on adults, leaving a gap in understanding the impact on young athletes. Jayanthi et al. (2019) suggest that youth sport specialization may raise the risk of psychological burnout.

The growing focus on specialized training raises concerns about potential long-term negative effects on young athletes, requiring further investigation. While the study’s findings are significant, its limitations, such as reliance on self-reported data from para-athletes and challenges in understanding certain questions, should be noted. Despite most participants finding the questionnaires ‘easy’, many faced difficulties with questions like ‘What is your type of disability?’ which slowed data collection and hindered participant recruitment.
